# Supplementary material for: Clients’ perspectives on the utilization of reproductive, maternal, neonatal, and child health services in primary health centers during COVID-19 pandemic in 10 States of Nigeria: A cross-sectional study
Source: PLoS One. 2023 Jul 20;18(7):e0288714. doi: 10.1371/journal.pone.0288714 (PMC10359015; doi:10.1371/journal.pone.0288714)
Supplement: S1 File — (DOCX) [file pone.0288714.s001.docx]

**Protocol 2 (Questionnaire)**

Client Perspectives

**Section 1: Socio-demographic Characteristics of the Respondents**

1. Name (Optional and may not be needed) ____________ State______________LGA____________
2. PHC in which the respondent is registered_____________________________________
3. Date of Registration ____________________ Registration Number (if applicable) _____________
4. Confirm Registration in this PHC before COVID-19 ________Yes_____________No
5. Age ____________(Years); Marital Status ____Married______Single _____ Separated _____Divorced ______widowed
6. Educational Level ______None; ______Primary; _______ Secondary; _____Tertiary; _____Other (state)
7. Religion ______None; _____Christian; _____Muslim; ________ Other (state)__________
8. Employment ________No _______Yes; Nature of Employment (State); ________________
9. No. of previous pregnancies ________ No. of Previous abortions _______ No. of living children________

**Section 2: Knowledge of COVID-19, experience and preventive behaviour**

1. Have you heard of Covid-19? 1) Yes______2) No ________
2. If yes, please tell us how you heard of covid-19 (state all that apply) : 1) radio; 2) TV; 3) internet/social media; 4) family members; 5 friends; 6) health worker; 7) others (state) _______
3. Tell us all the symptoms of covid-19 that you know_______ 1) fever 2)body aches 3) sneezing, 4) loss of smell or odor, 5) difficulty in breathing, 6) diarrhea, 7) cough (Respondent can select more than one)
4. Is it possible for a covid-19 patient to show no symptoms: 1) Yes____; 2) No_____ 3) Don’t know____
5. What steps do you personally take to prevent COVID-19 1) None ___ 2) social distancing ____ 3) Facemasks ___ 4) handwashing _____ 5) Others (specify)__________
6. Have you personally experienced COVID—19 or its symptoms? 1) Yes ___Yes 2) No _____
7. Do you know someone who has had or experienced COVID-19? 1) Yes____2) No____

**Section 3. RMNCH Services received before and after COVID-19 and disruption**

1. Which of these services have you received in this clinic before the pandemic? (Please state all services received): 1) Family planning; 2) antenatal care; 3) delivery care; 4) postpartum care; 5) Childcare; 6) Other reproductive health services (please state)
2. How often did you visit the clinic for these services before the pandemic? ________ Per week ____________ Per month
3. What difficulties did you experience with attending the clinic before the pandemic
4. No transportation 2) No money to pay for services 3) No drugs 4) No FP product 5) FP product of choice not available 6) Other (please specify) (Respondents to choose as many as apply)
5. After the COVID-19 started in March 2020, did you personally experience any problem with attending this clinic? ______ Yes; _____No
6. If yes, what were the difficulties
7. No transportation 2) Providers were not available 3) No drug 4) No FP product 5) Other (please specify) ………………………….. (Respondents to choose as many as apply)
8. How often did you visit the clinic for these services after the pandemic started? ________ Per week ____________ Per month
9. Did you miss any appointment in the clinic during after the pandemic started 1) yes 2)No
10. If yes, what appointment did you miss 1) Family Planning 2) Antenatal care 3) Delivery care 4) Postnatal care 5) Child immunization 6) Other (Please specify)
11. What did you do about the missed appointment 1) Used another facility 2) Did nothing 3) Used TBA 4) Other (Specify)

**Section 4. Experience of domestic violence before and after the pandemic**

1. Have you personally experienced violence (physical beating, rape, sexual harassment, etc.)? from your husband, or any male partner that you have? Yes________; No________
2. If yes, by who? 1)husband ____2) boyfriend_____3) any family member_____4) Teacher_____ 5) stranger ______6) Other (State)_______
3. If yes, did you experience the gender-based violence before or after the covid-19 started 1) Before____ 2) After______ 3) Both
4. If yes, did you attend this clinic for treatment from gender-based violence? 1) ______Yes; 2) __No
5. If No to question 17, do you know of anyone who has experienced gender-based violence? _____1) yes______2) No______
6. If yes to question 21, how is the person related to you? Please state_________
7. If yes to question 21, did the violence occur before or after the covid-19 started______1) before covid 19 started___________ 2) After covid-19 started_______

**Section 3: Stock-outs and RMNCH experiences pre-and post-COVID pandemic lockdown**

1. Was family planning one of the services you received in this clinic before and after COVID-19 started? ___Yes; ______ No
2. If Family planning, what method were you taking?______ Injectables; _____contraceptive pills;__Condoms; ________Emergency contraceptives_______ Others (pls state)______
3. Who advised you to use this method 1) husband_______2) friend______3) Relative and state relationship_______4) Others (state)_______
4. Did the supply of the method you used continue or diminish after COVID-19 started? 1) Continue; ____2) Diminish_____ 3) Stopped
5. If answer to 26 is diminish or stopped, what did you do? 1) Used another hospital 2) Did nothing 3) Called the PHC nurse for personal assistance 4) Other (Specify)
6. Did you experience pregnancy during the COVID-19 lockdown period? 1) Yes____ 2) No _______
7. If yes, was the pregnancy planned/wanted? 1) Yes______________ 2) No _________
8. If not planned or as unwanted, please explain what you did with the pregnancy 1) Continued the pregnancy 2) Terminated it____________
9. Did you attend the PHC for skilled pregnancy care before the pandemic lockdown? 1) Yes ___ 2) No ____
10. If Yes, which aspect of skilled pregnancy care? 1) Antenatal care ____2) Delivery___ 3) postnatal care___
11. What difficulties did you experience with attendance for skilled pregnancy care in the clinic before the pandemic 1) Provider not usually available 2) inadequate drug 3) no transportation 4) Cost too much 5) Other (specify)
12. Did you attend the PHC for skilled pregnancy care during the pandemic lockdown? 1) Yes ___ 2) No ____
13. If Yes, which aspect of skilled pregnancy care? 1) Antenatal care ____2) Delivery___ 3) Postnatal care___
14. Did you attend the PHC for skilled pregnancy care after the pandemic lockdown? 1) Yes ___ 2) No____
15. If Yes, which aspect of skilled pregnancy care? 1) Antenatal care____2) Delivery___ 3) Postnatal care___
16. Did you experience any difficulties when you continued with your attendance for skilled pregnancy care after the pandemic lockdown 1)Yes ____ 2) No ____
17. What difficulties did you experience? 1) Providers not available 2) Clinic not open 3) No drugs 4) Other (Specify) ____________ (Respondents to select as many as apply)
18. Did you try to immunize any of your children during the period? 1) Yes _______2) No_____
19. If the answer to the above question is Yes, did you experience any problems with immunization. Please, explain in your own words___________________
20. Apart from family planning and maternal and child health services, please tell us any other service you tried to obtain from the clinic during the pandemic ________________
21. Kindly explain in your own words your experience with trying to obtain such services 1) No transportation 2) Clinic closed 3) Provider not available Other (Specify)______________________

**Section 4: Recommendations**

1. COVID-19 pandemic has been very tough. What recommendations would you make to government and other responsible agencies for the provision of family planning and other reproductive health services during this pandemic? Please say this in your own words_______________

THANK YOU VERY MUCH
