# Supplementary material for: Clients’ perspectives on the utilization of reproductive, maternal, neonatal, and child health services in primary health centers during COVID-19 pandemic in 10 States of Nigeria: A cross-sectional study
Source: PLoS One. 2023 Jul 20;18(7):e0288714. doi: 10.1371/journal.pone.0288714 (PMC10359015; doi:10.1371/journal.pone.0288714)
Supplement: Attachment — Submitted filename: Rebuttal_Letter.docx [file pone.0288714.s002.docx]

July 21, 2022

Dear Editor,

Thank you for considering our manuscript PONE-D-22-19181 titled “Clients’ perspectives on the utilization of reproductive, maternal, neonatal, and child health services in primary health centers during COVID-19 pandemic in 10 States of Nigeria: A cross-sectional study”.

Please, see below our point-by-point response to the original reviewers’ comments, and editorial comments in the previous submission PONE-D-21-35477R1.

We have indicated how participant consent was obtained, documented, and witnessed.

Sincerely,

Professor Friday E. Okonofua

COMMENTS AND RESPONSE

Dear Editor,

We have addressed the issues you raised. Please, see our response below.

1. You indicated that you had ethical approval for your study. In your Methods section, please ensure you have also stated whether you obtained consent from parents or guardians of the minors included in the study or whether the research ethics committee or IRB specifically waived the need for their consent.

Response:

The research did not involve minors, thus, obtaining consent from parents or guardians was not applicable. This has been added to the ethical consideration in the methods section.

2. Please amend the title either on the online submission form or in your manuscript so that they are identical.

Response: This has been done. Thank you.

3. We note your current Data Availability statement: "The data are available on request from the UNFPA country Office, Nigeria"

Response: We have now deposited the associated dataset in an open repository (DOI 10.5281/zenodo.6128442).

EVIEW COMMENTS AND RESPONSE

Point-by-point response

Journal requirements

1. Please ensure that your manuscript meets PLOS ONE's style requirements, including those for file naming.

Response: This has been complied with.

2. Please amend your current ethics statement to address the following concerns:

a) Did participants provide their written or verbal informed consent to participate in this study?

b) If consent was verbal, please explain i) why written consent was not obtained, ii) how you documented participant consent, and iii) whether the ethics committees/IRB approved this consent procedure.

Response: The ethical approval statement has been amended to include how informed consent was obtained from the respondents.

3. You indicated that you had ethical approval for your study. In your Methods section, please ensure you have also stated whether you obtained consent from parents or guardians of the minors included in the study or whether the research ethics committee or IRB specifically waived the need for their consent.

Response: Minors were not involved in this study.

4. We note that you have indicated that data from this study are available upon request. PLOS only allows data to be available upon request if there are legal or ethical restrictions on sharing data publicly. For information on unacceptable data access restrictions, please see <http://journals.plos.org/plosone/s/data-availability#loc-unacceptable-data-access-restrictions>.

Response: The data associated with this paper are not deposited in an open repository because of ethical reasons.

In your revised cover letter, please address the following prompts:

a) If there are ethical or legal restrictions on sharing a de-identified data set, please explain them in detail (e.g., data contain potentially identifying or sensitive patient information) and who has imposed them (e.g., an ethics committee). Please also provide contact information for a data access committee, ethics committee, or other institutional body to which data requests may be sent.

b) If there are no restrictions, please upload the minimal anonymized data set necessary to replicate your study findings as either Supporting Information files or to a stable, public repository and provide us with the relevant URLs, DOIs, or accession numbers. Please see http://www.bmj.com/content/340/bmj.c181.long for guidelines on how to de-identify and prepare clinical data for publication. For a list of acceptable repositories, please see http://journals.plos.org/plosone/s/data-availability#loc-recommended-repositories.

We will update your Data Availability statement on your behalf to reflect the information you provide.

Response: This has been addressed.

REVIEWERS COMMENTS

Reviewer 1 (The reviewer tracked his/her comments in a PDF document)

**Abstract**

Comment 1: -these sentences is not clear please rephrase it.

Response: The result section of the abstract has been revised.

Comment 2: -this is the result section where the major finding of you work is mentioned, but you put the recommendation. So please see it again

Response: The recommendations that are presented in the result section are part of the results, not conclusive recommendations. They are recommendations from the respondents in response to a question.

Comment 3: -What things were raised by the women.

-which part take which recommendation to make it specific.

Response: The things raised by the women are the recommendations made by the respondents which are presented in the results section of the abstract.

**Introduction**

Comment 1: -First please mention in long sentence (Coronavirus )

Response: COVID -19 has been written as coronavirus

Comment 2: -please sensitized it and put in short.

-single references for such long paragraph is not recommended

Response: We were reporting the result of a previous study by us which prompted the current study. This is the reason for the single reference for the three sentences.

Comment 3: is the study is a comparative study???

Response: The “before and after” is used here to refer to before the coronavirus started and after it started not in the sense of a comparative study.

**Materials and Methods**

Comment 1: -this prevalence is high. why??? It may affect the result.

Response: The non-response rate of 23.7% - although COVID-19 protocols were observed during the data collection, more potential respondents than usual declined to be interviewed due to fear of contracting the virus.

Comment 2: -Is time is the only independent variable???

Response: The time care was received - before or after the pandemic started was the key independent variable for utilization of RMNCH services.

Comment 3: How do you manage these variables

Response: The effect of the potential confounding variables was controlled during the regression analysis by adding the variables in the regression.

**Results**

Comment 1: -please describe the response rate, how much of the participant were included in the study, why only 2930 is fully participate

Response: The response rate has been added, and the reason for the 2930 respondents reported in Table 1.

Comment 2: the frequency is 2927 not 2930

Response: The frequency for marital status was 2927 not 2930 because there were three of the 2930 respondents whose marital status was not recorded. This may be an omission from the data collectors or non-response by the respondent.

Comment 3: it is also 2929 not 2930 please see others since similar thing is expected

Response: The frequency for place of residence is 2929 not 2930 also due to variable-specific non-response. Place of residence was not recorded for one respondent – it may be an omission from the data collectors or non-response by the respondent.

We have added a note under the table to explain the discrepancy in frequency for some variables.

Comment 4: how many participant mention these and other challenges. please put the specific number

Response: The specific number of respondents who mentioned each of the difficulties and the corresponding percentage is in Table 4. We think that repeating the same numbers for each difficulty in the text will be unnecessary.

Comment 5: -in method section you have said that time is the only independent variables but here in table five there are other interdependent variable. how it could be??

Response: Time service was received was the key independent variable for utilization for RMNCH service. Experience of difficulty was another outcome variable with selected characteristics as the independent variables. This has been stated more clearly in the methods.

**Discussion**

Comment 1: -the discussion is highly shallow and needs serious revision

-It is not discussed with other related findings

Response: The findings have been discussed in relation to more previous studies

**Conclusion**

Comment: in which time is is limited before or during the pandemic

Response: COVID-19 pandemic here refers to after the pandemic started.

**Acknowledgement**

Comment: -your acknowledgment is very huge and boring to read

-is it acknowledgment or discussion????

Response: The acknowledgement is long because of the number of government and non-government agencies that facilitated the study at different stages. We have deleted the names of authors whose were also acknowledged.

**References**

Comment: -your references are small in number. so please search and add??

Additional studies have been identified and added.

Reviewer 2

General comments

Response: The responsiveness in the title has been changed to utilization to reflect the objective and results. Thanks.

Abstract

1. Combine Paragraph 1 & 2 (background & objective)

Response: This has been done.

2. Methods: The sentence is too long and hard to read.

• Mention the statistical software used and p-value.

Response: The sentences have been revised. The statistical software used and p-value have been included.

3. Results: The results are not well articulated hence hard to follow.

• What was the percentage of women who accessed RMNCH services before and onset of the pandemic?

• Which services were utilized more during the pandemic?

• What were the reasons for under-utilization of the services during the pandemic?

• Move the recommendations to the conclusion section

Response:

Many parts of the result section have been revised for clarity.

The analysis is essentially a comparison between the period before the pandemic and after the pandemic started. Table 2 in the results shows the number of women who used each of the services before and after the pandemic started.

The percentage and odds of utilization of each of the services before and after the pandemic started are reported in Tables 2 and 3.

The reasons for under-utilization of each service during the pandemic were reported as difficulties experienced in accessing the services. See table 4.

The recommendations that are presented in the result section are from the respondents, and it is a part of the objectives of the study. Recommendations arising from the results are presented in the discussion and conclusion sections.

Introduction

4. What is the national utilization of RMNCH services before and during the pandemic?

5. Provide a reference for “Even after the lockdown period, the number of women…..”

6. Use reproductive, maternal, neonatal and child health abbreviation (RMNCH) for uniformity.

Response: The national utilization for RMNCH services has been added. However, that is for 2018 when the most recent Nigeria Demographic and Health Survey was conducted. The national utilization is not for primary health care only; it includes utilization at the other levels of care – secondary and tertiary care.

To the best of our knowledge there is no published data on national utilization of RMNCH services after the pandemic started.

The reference for “Even after the lockdown period, the number of women…..” has been added. That evidence is based on our previous study.

We now use RMNCH as reproductive, maternal, neonatal and child health.

Materials & methods

7. Restructure this section: Study site, study design, study population, sample size, sampling procedures, data collection, outcome measures, data management and analysis.

Response: This has been done.

8. Paragraph 1: Replace clients with women

Response: client has been replaced with women.

9. Revise inclusion criteria 1 (the pandemic is still there)

Response: Thank you. This has been revised.

10. Sample size: Check the non-response calculation after 10% adjustment

Response: 10% of 384 =38.4, and 384+38.4=422.4, and we approximated to 422.

11. Data collection: Be concise in this section. The information is there but the presentation is poor.

• Paragraph 1 is unnecessary in this section (delete)

Response: This has been done.

• Restructure the information: data collection period, data collection tools, data collection procedures

Response: The information has been restructured.

• Were the data collection tools pretested?

Response: Yes, the questionnaire was pretested. A sentence on this has been added. Thank you.

• Who collected the data?

Response: The data were collected by trained data collectors who were recruited for the study. This is stated in the data collection section.

• Move information on how respondents were identified to sampling procedure section

Response: This has been done.

• Was the questionnaire or protocol the data collection tool? Replace “protocol contained basic questions with questionnaire contained….?

Response: The protocol is the questionnaire and the data collection tool. Protocol has been replaced with questionnaire.

12. Ethical consideration: the target group is women but nurse/midwife are mentioned in this section. Did the women consent to participate in this study? Also the delete “the objective was addressed using a questionnaire survey…….”

Response: This has been done. Thank you.

Results

This section is poorly written and hard to follow.

Response: Some revision has been made for better flow.

13. Check table 2: The target group was women who received RMNCH services before the pandemic. What was the number of women who utilized any of the RMNCH services before and after the pandemic? The assumption is before the pandemic 2930 women (100%) utilized the services. Then give utilization per specific RMNCH service.

Response: 2930 was the achieved sample size for the study out of the expected 3840, and all of them utilized one or more RMNCH services before and after the pandemic started. Each woman did not use all the five RMNCH selected services. However, not all the 2930 responded to all the questions on utilization of each service. This accounts for the shortfall in the total before the pandemic, and after the pandemic started.

The utilization for each service before the pandemic and after the pandemic started is what is presented in Table 2. The total number of responses for all the services before pandemic was 2,717, and 2461 after the pandemic started.

14. How did you differentiate challenges before and during the pandemic? Table 4 does not show challenges before and during the pandemic.

Response: Data were collected on challenges (difficulties) before and after the pandemic started. See rows 2 and 4 in Table 4 for presentation of the results.

15. There are two table 6 (logic regression and Recommendation)

Response: This has been corrected. Thank you.

Discussion and conclusion

16. How do the results compare to other studies?

17. The discussion should be linked to the results.

Response: The discussion has been updated to reflect a link to more studies.
