# Supplementary material for: Clients’ perspectives on the utilization of reproductive, maternal, neonatal, and child health services in primary health centers during COVID-19 pandemic in 10 States of Nigeria: A cross-sectional study
Source: PLoS One. 2023 Jul 20;18(7):e0288714. doi: 10.1371/journal.pone.0288714 (PMC10359015; doi:10.1371/journal.pone.0288714)
Supplement: Attachment — Submitted filename: Response to Reviewers.docx [file pone.0288714.s003.docx]

May 10, 2023

Dear Editor,

Thank you for considering our manuscript PONE-D-22-19181 titled “Clients’ perspectives on the utilization of reproductive, maternal, neonatal, and child health services in primary health centers during COVID-19 pandemic in 10 States of Nigeria: A cross-sectional study” for publication.

Please, see below our point-by-point response to the editor’s and reviewers’ comments.

Sincerely,

Professor Friday E. Okonofua

**Response to Editor’s comments**

Journal Requirements

1. Please ensure that your manuscript meets PLOS ONE's style requirements, including those for file naming. The PLOS ONE style templates can be found at

Response: The PLOS ONE style has been followed.

2. Please provide additional details regarding participant consent. In the ethics statement in the Methods and online submission information, please ensure that you have specified whether: 1) whether the ethics committee approved the verbal/oral consent procedure, 2) why written consent could not be obtained, and 3) how verbal/oral consent was recorded.

Response: Verbal consent was obtained since the data collection was by computer-assisted personal interviewing, and a statement of information and consent was a part of the questionnaire. This has been explained in the ethical approval section.

**Response to Reviewer’s Comments**

Reviewer #1: -What was the basis or criteria of selection of the 10 communities in each LGA used as study sites?

Response: The ten communities were selected purposively based on location of a PHC in the community.

-Was ethical approval for the study obtained from any Health Research and Ethics Committee (HREC)? Ethical approval is different from permission from health ministries and centers to perform a study. State clearly the HREC that approved the work with the approval number.

Response: Approval to conduct this study was obtained from the various Ministries of Health in each State, and from the Research Ethics Committee, College of Medical Sciences, University of Benin, Nigeria #CMS/REC/2020/092 dated October 4, 2020.

As regards the inclusion of women who registered as having received clinical services in the clinics per state before and after the pandemic started. It is important to state the range of years before the pandemic that was targeted in this study? Were women who had accessed these services 10years ago also identified and recruited?

Response: There are three selection criteria. The second one is women receiving RMNCH and family planning services in the PHCs indicating that they were also using these services at the time of interview.

-Table 2 claims to describe “the pattern of utilization of RMNCH services pre- and post-covid”. How did you make up for/exclude women who did not need some of these services before or after the pandemic? It is important to differentiate women who did not require the service from those who needed it but couldn’t access it. Reasons why some women in the study may not have needed antenatal care before the pandemic could be because they were not pregnant before the pandemic. A woman who utilized post-natal services before the pandemic may not have needed it after the pandemic because she had no delivery after the pandemic. How did you ascertain that “no-need for a service” has not influenced the pattern depicted in Table 2?

Response: The study population were women who were using a PHC in their community for any of RMNCH services. It excludes women who had no need of the RMNCH services.

-Clearly state what confounders were adjusted for in Table 3?

Response: The confounders adjusted in the regression presented in Table 3 were listed in the methods section under variables and measures. They are respondents’ age, marital status, the highest level of education, religion, number of previous pregnancies, number of living children, knowledge of COVID-19, place of residence, and State of residence.

- A copy of the study questionnaire should be included as a supplementary file to the reviewer to help assess the work better.

Response: A copy of the questionnaire has been included as a supplementary material.
